# Better Healthcare Together: Co‐Designing a Framework for Health Service Co‐Design

**DOI:** 10.1111/hex.70784

**Published:** 2026-07-28

**Authors:** Adrienne M. Young, Anja Christoffersen, Dale Trevor, Suzanne Stringer, Tim Cudmore, Leonie Sanderson, Gillian Nasato, Lisa Anemaat, Katrina L. Campbell

**Affiliations:** ^1^ Dietetics and Food Services Royal Brisbane and Women's Hospital Brisbane Queensland Australia; ^2^ Australian Frailty Network, Frazer Institute The University of Queensland Brisbane Queensland Australia; ^3^ Consumer Representative Metro North Health and Queensland Health Brisbane Queensland Australia; ^4^ Champion Health Agency Mt Coolum Queensland Australia; ^5^ Cudmore Consulting Upper Cooomera Queensland Australia; ^6^ People Tech Revolution and The Ageing Revolution Brisbane Queensland Australia; ^7^ Queensland University of Technology and Metro North Health Nursing & Midwifery Academy Brisbane Queensland Australia; ^8^ Surgical Treatment and Rehabilitation Service (STARS) Education and Research Alliance Metro North Health and The University of Queensland Brisbane Queensland Australia; ^9^ Queensland Aphasia Research Centre, School of Health and Rehabilitation Sciences The University of Queensland Brisbane Queensland Australia; ^10^ Healthcare Excellence and Innovation Metro North Health Brisbane Queensland Australia; ^11^ Centre for the Business and Economics of Health The University of Queensland Brisbane Queensland Australia

**Keywords:** co‐design, community participation, health planning, health services, health services research, user‐centred design

## Abstract

**Introduction:**

Co‐design in health services is increasingly recognised as essential for creating services and policies that address patient and community needs. However, genuine co‐design can be challenging, with existing toolkits often catering to professionals, reinforcing power imbalances and limiting community leadership. This paper aims to present a novel co‐design framework for health services and describe the co‐design process taken in its development.

**Methods:**

The co‐design process involved a series of engagement activities (online forums, focus groups, discussions and workshops) with health consumers, health professionals and researchers within a metropolitan health service in Queensland, Australia. This process, co‐led by a clinician researcher and health consumer, explored the implicit meaning of co‐design and the barriers and enablers to co‐design within the local setting, and the strategies required to support operationalisation of co‐design. These data were combined with research literature, existing frameworks and lived experience to iteratively co‐design the online framework.

**Results:**

Eight focus groups, forums and workshops across five hospitals and multiple community health services involved 128 individuals who contributed their professional and lived experience with co‐design to inform the development of the co‐design framework. The resultant ‘Better Healthcare Together’ framework includes three main components: (1) before you start co‐design, (2) the co‐design team and (3) the co‐design process. A key feature of the framework is its deliberate focus on power, inclusion, equity, and bringing together diverse lived and professional experience to solve healthcare problems.

**Conclusions:**

This co‐design framework provides a flexible approach to consumer partnerships, with reflections on the process highlighting the relational nature, the need to avoid expert mindset and the evolving landscape of consumer engagement. While widely used, the framework alone may not build true health system co‐design capability due to the need to address systemic and organisational barriers to co‐design.

**Patient or Public Contribution:**

Members of the public contributed to this work across all stages of this project, including as project co‐leads and co‐researchers, members of the steering committee, workshop co‐facilitators, and co‐design participants. They are also co‐authors on this paper.

## 1 Introduction

Co‐design is increasingly used in the health sector to design health services and policies that better address patient and community needs and concerns. Whilst a single common definition of co‐design does not exist, a recent review of all available literature has proposed the following definition: “a collaborative and inclusive design approach that actively involves diverse stakeholders in the design process to address a specific problem, ensuring that varying degrees of participation throughout different stages lead to innovative and relevant solutions that meet the needs and expectations of all participants [1]. ” Within the health co‐design context, what is missing from this definition is the explicit inclusion of health consumers (people with lived experience of the healthcare context, i.e., those who have used, or may potentially use, health services, or are carers for patients using health services [2]) as key participants and potentially leaders of the co‐design process. Involvement of health consumers in health system redesign and service improvement has been referred to as the new “Zeitgeist” [3] and is a movement that is now not only seen as ‘ideal’ but necessary to achieve a new moral era of healthcare [4]: an era where the voices of people served are heard [4] and participation in healthcare at all levels of the system is respected as a fundamental principle of human rights [5]. Patient and public involvement (PPI) in health service design and improvement can also encourage democratic decision making, public accountability and transparency [6, 7], overall increasing trust in the healthcare system. As a result, co‐design is now a requirement mandated in health service standards and policies in countries including Australia, England and Canada [2, 8, 9]. However, operationalising genuine co‐design within health systems can be challenging. In fact, some researchers have identified a risk of harm (or at best, wasted time and effort) when co‐design is approached in a tokenistic manner (e.g., risk of re‐trauma, reinforcing health inequities) [3, 10].

Many toolkits and online resources have been developed to enable co‐design (e.g., Co‐design Capability and Conditions Framework [11], Experience‐Based Co‐design Toolkit [12]); however these were largely created for designers, researchers or health service staff to lead a process where they ‘engage’ with health consumers, rather than enabling them to have some control over the process. This is evident in these frameworks in the lack of roles articulated for consumers in deciding how the process of co‐design will be enacted [13, 14] and in the absence of guidance about strategies to support power sharing [15]. Adding to the confusion for people new to co‐design are the many different definitions for the term ‘co‐design’ and a lack of clarity about how it is different to other ‘co’ approaches (such as ‘co‐production’ and ‘co‐creation’) and forms of PPI (also called ‘consumer engagement’ and ‘citizen or public involvement’) [16]. Developing a framework for co‐design using a co‐design process involving people with professional and lived experience may allow for the generation of shared understanding, language and approach to enable more genuine execution of co‐design.

The aim of this paper is to present a novel co‐design framework for health services called ‘Better Healthcare Together’, which was co‐designed by and for health consumers, health service staff and researchers. We describe our process, outline the framework and resources for others to use and/or adapt, and share our reflections as a team of health consumers and health services staff new to co‐design.

## 2 Materials and Methods

### 2.1 Study Design

The framework co‐design process involved a series of engagement activities (online forums, focus groups, discussions and workshops) over 12 months in 2021‐22 using a collaborative and creative generative process, leveraging our collective experience as health consumers and health service staff (including clinicians, researchers, designers, community engagement professionals and health service managers). Table 1 outlines the phases of the study, informed by existing co‐design process models by KA McKercher [17], groups such as The Auckland Co‐Design Lab [11] and the Experience Based Co‐Design toolkit [12]. Reporting of the consumer involvement in this paper is guided by Guidance for Reporting Involvement of Patients and the Public (GRIPP2) [19] (Supporting Information: Material 1) to support transparency of PPI practice.

**Table 1:** An overview of the co‐design process and engagement activities guided by co‐design phases from existing frameworks [11, 12, 17, 18].

Phase	Purpose	Activities	Participants
Engage and align	Define project success Decide who needs to be involved Define co‐design	Development of funding pitch and project plan Initial steering committee meeting Online workshop (topics: co‐design definitions, barriers to co‐design) Online forum	Co‐leads Steering committee Consumers Health workers Health leaders Researchers
Explore and connect	Gather diverse perspectives on co‐design	Online forum Group and individual meetings (online or in‐person) In‐person workshops (topics: power, equity)	Co‐leads Consumers Health workers Health leaders Researchers Designers
Imagine and decide	Decide on framework structure, features and content	In‐person workshops (activities: developing the ‘recipe’ for co‐design) Steering committee meetings	Co‐leads Steering committee Consumers Health workers
Create and test	Develop and test framework content Develop and test community of practice	Prototype online framework Initial testing by steering committee Co‐write section on ‘*Equity and Co‐design*’ with Aboriginal and Torres Strait Islander team Community of practice pilot programme (online, 1 h per month for 6 months) Framework testing by novice co‐designers	Co‐leads Consumers Health workers Designers Researchers Steering committee
Co‐implement and co‐evaluate	Implement framework and community of practice Evaluate use and experience	Website go‐live Continuation of Community of Practice Collect usage data	Web publishers Co‐leads Consumers Health workers Researchers Designers
Share and celebrate	Disseminate the framework to diverse audiences Acknowledge all contributors Celebrate the end‐product and process	In‐person launch event Presentations – internal (health service executive meetings, forums, research symposia, consumer advisory groups) and external (e.g., Health Consumers Qld, Health Translation Qld, Patient Experience Healthcare Week)	Co‐leads Steering committee Everyone involved

*Note:* While the process is presented linearly, in practice the process was iterative, with movement back and forth during ‘*Explore and Connect*’ and ‘*Imagine and Decide’* phases, in response to topics identified during the engagement activities.

### 2.2 Setting

The setting for this co‐design framework is a metropolitan health service district in Queensland, Australia that serves a population of nearly 900,000 people over a geographical area of around 4100 square kilometres. At the time of this project, the health service included six hospitals and numerous community health services (including oral health, mental health, public health and child health) and employed around 19,000 staff.

The local need for the framework was identified in 2019‐20 by a group of health consumers and health service staff, who identified a grass‐roots interest in co‐design, but that people did not know what co‐design was or how to do it practically. Before commencing the framework co‐design process, a literature review and online environmental scan were undertaken to identify existing frameworks. We concluded that, while there are many existing frameworks, none were fit for purpose; that is, no frameworks were designed to be consumer facing and existing frameworks were either not accessible for everyone (e.g. behind organisational intranet sites) or not designed for beginners to co‐design. Importantly, no existing frameworks were identified that were designed with and for health consumers or that adequately considered and supported collaboration between health consumers and health service staff where they made decisions together as equals [20]. Further, no frameworks were identified that equipped all potential users with the same knowledge and resources to lead the co‐design process which we identified as essential for co‐design success and power sharing. Funding to develop the framework was obtained through an internal funding scheme aimed to support the design and implementation of innovations aligned to the priorities within the health service.

### 2.3 Definition of Co‐Design

Given the potential for confusion about terms and definitions, it was important that we defined early in our process what was meant by the term ‘co‐design’ within our health service. Participants at a workshop and online forum (19 health consumers and 63 health service staff) worked together in small teams to create their own definitions of co‐design (described further in Supporting Information: Materials 2). Using insights from this process, our team co‐designed the following definition to guide the development of our co‐design framework:

Co‐design is a process where people with professional and lived experience partner as equals to improve health services by listening, learning and making decisions together.

Deliberate wording choices were made within this definition to reflect key concepts raised by workshop and forum participants. These included power sharing, authentic partnerships, doing things together, and a focus on relationships above outcomes or the design methods, as well as the strong preference to describe co‐design participants as people and not based on their role. A suggestion from participants was to develop a short engaging catch‐phrase for co‐design to help people to understand co‐design. A consumer member of the steering committee suggested the catchphrase ‘Better Healthcare Together’ which was endorsed by the group and subsequently adopted as the name of the co‐design framework.

### 2.4 Co‐Design Approach

The project was co‐led by a health consumer (AC) and clinician‐researcher (AY) who made all decisions about the project together. Consumer co‐lead (AC) has lived experience of disability, was a full‐time business student and had 5 years of experience in consumer engagement, participating in committees at state and national level, including as Deputy Chair of the hospital's Consumer Advisory Group. Health service co‐lead (AY) had 15 years of experience working as an allied health professional within the health service, including 3 years of experience involving consumers in research and quality improvement projects. The consumer co‐lead position was remunerated for regular hours (4 h per week). The project co‐leads had not previously worked together.

Project co‐leads were supported by a steering committee who had an advisory function and met monthly throughout the project. This committee, co‐chaired by project co‐leads, included four consumers (three experienced, one new to consumer engagement) who were remunerated for attending, two consumer engagement staff, six health service staff (two clinician/researchers; four managers) and one person external to the health system. This external person with community engagement and design experience was specifically sought for the committee to provide an external perspective and act as a provocateur [17].

As novice co‐designers, our approach to co‐design was influenced by previous positive engagement experiences, existing frameworks and resources [11, 12], equity and trauma informed practice [18, 21], and a commitment to true collaboration. In particular, we were influenced by the work of designer KA McKercher [17]. Foundational to our approach were the following principles: (a) decisions would be made collaboratively between health consumers and health service staff at each stage of the process, (b) we would additionally engage widely to inform, consult with and involve diverse health consumers and health service staff, whilst supporting opportunities for consumer empowerment, and (c) we would adopt a co‐design mindset. This means that we strived to elevate lived experience, practise curiosity, offer generous hospitality, be in the grey (i.e. embrace uncertainty), learn through doing, and value many perspectives [17].

### 2.5 Sampling

Participants in the development process included health consumers and health service staff (clinicians, managers, quality improvement officers, researchers). They were invited to attend a range of engagement activities through either an open or targeted invitation, depending on the purpose of the activity (Table 1). For the online forum, an open invitation was extended across the health service to anyone interested in or experienced in co‐design. This invitation was distributed through health service bulletins, relevant email lists (e.g. from the research office to all researchers working in the health service; from the consumer engagement team to all health consumers on their distribution list) and word of mouth, with people encouraged to forward the invitation to their peers. This was followed by targeted workshops and meetings, with individuals or groups, who were either identified by the steering committee or others as key informants (e.g. the health services’ Aboriginal and Torres Strait Islander Leadership Team).

### 2.6 Data Collection and Analysis

Data were collected through a series of workshops, forums, and meetings with groups and individuals (Table 1). All activities were co‐planned and co‐facilitated by the project co‐leads, with input from design experts and peer mentor (LA). An exception to this was the workshop on the topic of power and co‐design, which was planned and facilitated by our external steering committee member (LS) to address any potential or perceived power imbalance between project co‐leads and participants. Each engagement activity included discussion questions and activities tailored to the purpose and the audience of the activity (Table1). All project activities took place between July 2021 and June 2022, during periods of pandemic‐related public health measures limiting visitors to health services and size of public gatherings. Therefore, many activities were undertaken online via Zoom or Microsoft Teams. Data were collected from all activities in the form of audio‐recordings, participant‐driven sticky notes (digital and paper‐based), online whiteboards (participant and facilitator contributions), facilitator field notes and meeting minutes. Together the project co‐leads reviewed the data after each activity. An implementation science framework was used as an analytical framework to identify barriers and enablers to conducting co‐design in our health service. In line with Elo and Kyngäs’ deductive content analysis approach [22], the i implementation determinants from the integrated Promoting Action on Research Implementation in Health Services framework (i‐PARIHS) [23] formed a structured categorisation matrix against which data were coded, facilitating the examination of how local contextual factors influenced co‐design implementation (Supporting Information: Table S1). These data, alongside published literature and existing co‐design frameworks and resources [11, 12, 17, 18], were presented and discussed with the authorship group at regular meetings. Together the group made decisions about the framework components and the content within these, based on the needs identified during the engagement activities. Recognising the substantial body of existing co‐design and implementation resources, we developed an online prototype that integrated content from published sources wherever possible. This approach enabled the framework to act as a central access point to existing guidance and templates, maximising accessibility while minimising duplication. The online prototype was tested by steering committee members and novice co‐designers, with their feedback then used to refine the prototype, resulting in the final framework.

### 2.7 Ethical Considerations

The project plan was reviewed by the hospital ethics committee and deemed to be exempt from formal review as it did not meet the definition of research (EX/2021/QRBW/80178). All consumer engagement was remunerated in accordance with health service policy, with the exception of the online forum.

## 3 Results

Across 12 months, we engaged with 128 individuals, including 28 health consumers and 100 health service staff (clinicians, managers, quality improvement officers, researchers) to co‐design the co‐design framework, across eight formal engagement activities and as part of the steering committee. Data from the online forum (our largest engagement activity; *n* = 68) demonstrated diversity in contributors, including people from culturally and linguistically diverse backgrounds (*n* = 7), Aboriginal and Torres Strait Islander (i.e. First Nations Peoples of Australia) (*n* = 4), LGBTQIA+ (*n* = 3), people with a disability (*n* = 2), and both youths (under 25 years, *n* = 5) and older people (over 65 years, *n* = 1). As two white, cisgender females co‐leading this project, we leveraged diverse networks within our personal communities and the health service to seek further diverse perspectives, particularly from people who identified as Aboriginal and Torres Strait Islander, from a culturally and linguistically diverse background or from the LGBTQIA+ community. This resulted in additional informal conversations with numerous individuals and groups to inform the co‐design of the framework.

Based on insights from these engagement activities, the ‘Better Healthcare Together’ co‐design framework was developed, consisting of three main components: (1) *Before You Start Co‐design*; (2) *The Co‐design Team*; and (3) *The Co‐design Process*. A distinguishing feature of our framework is the explicit emphasis on pre‐conditions and team composition alongside co‐design processes. While these elements may be reflected within existing frameworks, they are often less prominent than guidance around the design process and methods. People told us that a co‐design framework should not be prescriptive, as the process needs to be tailored to the context, aims, funding, skill set of the people involved and timeframes for each specific project. Therefore, our framework includes general information, with prompts and templates to get co‐designers started.

The framework is supported by our co‐design definition, case studies, additional resources (e.g., websites, learning opportunities), and specific advice for co‐designing with Aboriginal and Torres Strait Islander Peoples and communities. This advice was developed by the health service's Aboriginal and Torres Strait Islander leadership team and was included as its own section in recognition of greater inequities in healthcare access and outcomes and their specific preferences and needs when co‐designing [24].

## 4 Before You Start Co‐Design

Participants told us that there are necessary pre‐conditions required to support co‐design. Despite the best of intentions, projects may be called co‐design when they are not or cannot be (due to time and resource constraints), potentially breaking the trust necessary to form long‐lasting and productive relationships. Our framework includes a *Fundamentals for Co‐design Checklist* (Box 1) to prompt people to consider whether their project is suited to co‐design (i.e., where there is genuine opportunity for consumers to genuinely partner and equally influence decision making) and to guide them to consider what needs to be addressed so that they can genuinely co‐design.

Box 1 **Fundamentals for co‐design checklist.**

**Table:** 

Prompt question	Response option supporting co‐design
Has a decision been made about a solution?	No
Do the community/consumers see the problem, challenge or opportunity being addressed as a key issue?	Yes
Can you secure the resources (e.g. funding) and commitment to deliver your project/solution?	Yes
If funds are committed already, is there a budget for consumer/community engagement?	Yes
Is there an impartial and skilled facilitator available to assist in the project if needed?	Yes
Is there a strong ‘expert mindset’?^a^	No

Consistent with research literature on co‐design, power and equity were key themes throughout our conversations about co‐design with health consumers and health service staff. Strategies to share power and reduce imbalances in power are woven into the fabric of our co‐design framework, informed by a workshop with health consumers and health service staff to ideate strategies to address power imbalance (examples in Box 2).

Box 2 **Strategies to reduce power imbalance in co‐design.**

**Table:** 

1. Funding and time: Having sufficient funding to pay health consumers shows the value placed on people's time and contributions and increases the possibility for people to participate more frequently, as needed and desired. 2. Decision making: Starting a project with a ‘clean slate’ without pre‐decided aims and probable outcome of the project immediately increases the power for health consumers to influence the project in a meaningful way. 3. Facilitation: Handing over the control of facilitating engagement activities to an external party can help to balance power, reduce bias and manage conflicting perspectives. 4. Equity: Acknowledging inequity and developing inclusive engagement methods can help to include and elevate the perspectives of marginalised communities and groups. 5. Physical environment: Choosing spaces that are safe and accessible to all people involved can reduce power imbalance and provide psychological safety. Often this means that co‐design processes need to happen outside of the healthcare environment.

## 5 The Co‐Design Team

The people involved in the co‐design process were identified as a ‘key ingredient’ for successful co‐design. The notion of a ‘co‐design team’ was established which included a small group of key people with lived experience, professional experience and external perspectives who have defined (and paid) roles to lead the process. They are the group that make key project decisions, drawing on perspectives from the broader community obtained through focused engagement and activities. Considerations in forming the co‐design team are outlined within the framework and fundamentally come back to sharing power within the team through valuing all team members, believing that everyone has something to learn (and occasionally unlearn) and something to teach, and recognising the need to build relationships and care for people through the process. The framework encourages the team to consider how they can create opportunities for health consumers to build skills by co‐facilitating meetings and engagement activities, undertaking interviews with other consumers, or in project management; likewise, encourage health consumers to contribute to the project beyond their lived experience.

The framework supports a trauma‐informed approach to co‐design. Health consumers come to co‐design health services and policies because they have had a lived experience with the healthcare system, which almost always involves some degree of trauma. For some people, this experience can be highly traumatic and may exist on a background of pre‐existing trauma particularly when partnering with people from marginalised groups. It is possible that health service staff may have also experienced traumatic events; therefore, caring for themselves and others during the co‐design process is paramount. Considerations for engaging with lived experience are included in the framework, including prompts to consider asking how people want to contribute and providing multiple ways for people to do this. Shared disclosure is also suggested to allow all team members to experience and demonstrate vulnerability in sharing personal experiences, building trust in team members. Offering formal supports such as post‐activity debriefing sessions with a counsellor or psychologist are also encouraged for all members of the co‐design team. Having a skilled facilitator was identified as a ‘key ingredient’ within the co‐design team, particularly where there is power imbalance, history of conflict, or divergent/ingrained viewpoints. This person needs to always remain impartial, maintaining a professional boundary between the process and the emotion to support the group to achieving their purpose. In one workshop, a health consumer raised the issue of accountability for team members, when co‐design often exists outside of traditional systems of supervision and management. As a result, the framework includes a Peer Performance Review that can be used by the team to hold team members accountable to the process, mindset and their role, assess team performance, track progress, anonymously provide feedback, and have balanced information to assess when a team member may not be the ‘right fit’.

## 6 The Co‐Design Process

**Figure 1:**
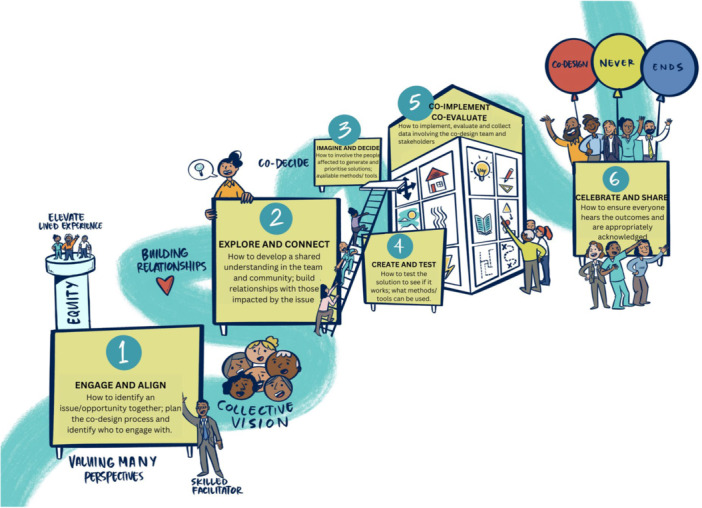
The co‐design process from the ‘Better Healthcare Together’ co‐design framework.

Guided by our community engagement, we adapted existing resources [11, 12, 17] to propose a model for the co‐design process (Figure 1). Wherever possible, users are directed to existing design resources, acting more as a starting point for the design journey than a comprehensive design tool. We recognised that many novice co‐designers might come to the framework looking for the steps without realising the importance of the pre‐conditions and team; therefore, prompts are embedded within the process to consider concepts such as power sharing and engaging lived experience. The co‐design process is presented as a linear but winding process, with prompts to link back and forth between the steps to support flexibility in its application.

### 6.1 Implementation of the Framework and Outcomes to Date

The ‘Better Healthcare Together’ framework was published online in late 2022 and the sustained use of the framework (> 36,000 page views and > 8000 users between March 2023 and December 2024, source: Google Analytics) confirms an interest and need for co‐design knowledge and skill development. The framework has been used to guide local projects within our health service [25, 26], with wider adoption as evidenced through framework citations in research publications from external teams [27], podcasts [28] and blog‐posts [29, 30], and informing the development of and/or included within new consumer engagement frameworks for public health services [31, 32].

## 7 Discussion

Through a collaborative generative process, a co‐design framework was co‐designed by health consumers and health service staff providing a common approach for health service improvement and research. Whilst there are many co‐design frameworks available in health and research [33, 34], the ‘Better Healthcare Together’ framework is unique in its development approach (co‐led by health consumers and guided by lived experience of co‐design) and its intended audience (both health consumers and health service staff). The methodological choices made during framework development likely shaped the framework itself. First, the decision to co‐lead the project through a consumer‐health service partnership placed issues of power sharing, relationships and equal decision‐making at the centre of the development process and may explain the prominence of these concepts throughout the final framework. Second, the use of i‐PARIHS as a deductive analytical framework encouraged consideration of contextual, organisational and individual determinants of implementation, contributing to the inclusion of sections focused on pre‐conditions and team readiness rather than solely the co‐design process itself. Finally, the decision to prioritise framework development and creating a practical output over comprehensive qualitative inquiry meant that the resulting framework was influenced by pragmatic design needs and iterative stakeholder input, rather than being derived from formal thematic analysis. Consequently, the framework should be understood as a co‐designed synthesis of stakeholder experience, implementation science concepts and existing evidence rather than a framework generated exclusively from qualitative data.

In contrast with existing co‐design frameworks [34], our framework includes strategies to support power sharing and trauma informed approaches [18, 21]. Health services are traditionally hierarchical with a clear chain of command [35], with power imbalance present between staff groups (e.g. administration and clinicians) and disciplines (e.g. doctors and nurses). Significant power imbalance exists in the patient and provider relationship, even more so when there are other ways that a patient has less power (due to race, ethnicity, age, disability, religion, sexuality, education and socioeconomic status) [17]. This power imbalance persists when a patient becomes a health consumer [36], requiring the careful and deliberate implementation of strategies within the co‐design process to attempt to balance power. The need to embed a ‘model of care’ within co‐design processes is being increasingly recognised [17]. However, outside of mental health settings, the implementation of trauma informed practices in health services is rare [37], posing a risk that co‐design in health services could inadvertently traumatise (or re‐traumatise) consumers where the trauma‐informed practices to promote safety, choice, autonomy, collaboration and respect are not embedded within the process. The prominence of power sharing throughout our engagement activities reinforces growing recognition that power is not a peripheral consideration in co‐design but a fundamental determinant of whether participation is experienced as meaningful or tokenistic. Similar concerns have been identified by others [15, 36], who describe persistent structural and relational power imbalances between health services and consumers despite organisational commitments to partnership. Our findings suggest that practical mechanisms for sharing power, such as shared decision‐making, remuneration, external facilitation and transparency regarding influence over outcomes, may require greater attention within co‐design frameworks than is currently provided.

Our co‐design process model also differs slightly to existing process models. Firstly, we introduced a step focused on ‘*Co‐implementation and Co‐evaluation*’ to capture insights from participants that there should be a continued partnership with consumers beyond the design phase. This finding suggests that participants viewed partnership as an ongoing commitment rather than a discrete design activity, aligning with implementation science perspectives that increasingly recognise end‐users, including health consumers, as active partners throughout implementation and evaluation [38]. The absence of practical guidance for co‐implementation remains an important gap in the literature, suggesting an area where future co‐design research and framework development may be particularly valuable [39]. Secondly, there is an emphasis on ‘*Sharing and Celebrating*’, for both the outcomes of the co‐design and also on the process that was taken, reflecting the consumer experience of an absence of feedback about how their input influenced a process or project and a lack of acknowledgement of their contributions which are often invisible.

Numerous barriers to conducting co‐design in health services have been identified through this project and across the published literature [40, 41, 42]. Whilst a framework can address barriers related to the concept of co‐design (through providing a common language, process and tools) and the individuals involved in co‐design (through knowledge development), we acknowledge that a framework alone is unlikely to build true health system capability for co‐design. This will require multi‐level strategies to overcome barriers related to organisational culture, processes and funding. One supporting strategy that we have implemented alongside the framework in our health service is a community of practice to provide peer‐led mentorship and opportunities to learn about, share experiences and practice co‐design, identified as key to building capability in consumer partnerships [43]. Consistent with emerging capability frameworks [43], our findings suggest that effective co‐design requires more than knowledge of methods alone and depends on organisational conditions, skilled facilitation and support for consumer‐health service partnerships.

As a new consumer‐health services staff partnership brought together to co‐design this co‐design framework, we have reflected on what we learned through the process of ‘doing’ true co‐design for the first time. We learned that co‐design is *a relational process*, evident in the long‐lasting relationship developed between the lead authors (having since co‐authored seven papers and received research funding on six grants together), as reported in other co‐design research [44]. Importantly, relationships were built with the broader co‐design team and community, instilling a feeling of shared ownership by all involved in developing this freely available online framework. We saw the value in a*voiding expert mindsets*, coming to the task of co‐designing the framework with a genuine drive to learn from others and a curious mindset open to exploration of ideas and ‘trial and error’. Rather than approaching it with a ‘right way of doing things’ mindset, we instead adopted an ‘are we doing this right?’ attitude to welcome feedback and refine how ideal, genuine co‐design should be experienced. We learned that *balancing power* is the most critical issue to address for successful co‐design, though is hard to achieve within the constraints and requirements of health services. The need to submit project plans, budgets and timelines upfront can limit the ability to offer a ‘blank canvas’ that is often needed for true co‐design from the very beginning. However, the project plan and health service leadership enabled enough freedom to adapt as we progressed, which contributed to authentic co‐design during the process. Remuneration and employment had impacts on power, as the clinician‐researcher lead continued to be employed inside the health service to deliver the project, whilst the consumer lead remained an ‘outsider’. While it is crucial for consumers to remain independent and outsiders to the system to voice a genuine consumer perspective, systems for remuneration and access to information broadened the inequities. Without consumers having choice in how they were engaged, it became apparent that there were structural and hierarchical barriers to address to ensure project co‐leads were on a ‘level playing field’. Strategies to overcome these barriers may include independent contractor arrangements for consumers that do not compromise other consumer representative roles, enable equal hours worked if the consumer chooses and offer the ability to negotiate the rates and processes for payments that may not be available through typical consumer honorariums. Finally, we learned the power (and the conflicts) in *bringing your whole self* to the co‐design process, recognising people's skills and expertise beyond only our lived and professional expertise. This not only supported relationship building and safe spaces for shared disclosure but created space for consumers to adopt additional roles and responsibilities. There can be tensions created by this, for the consumer in discerning when to contribute and when to be an unbiased conduit for others’ perspectives, and for health service staff being unsure when to bring their own lived experience to a co‐design project, and when to ‘bracket’ this and only bring their professional perspectives.

There are limitations in the process taken and the resultant framework that we want to highlight. Whilst we strived for wide consultation to bring diverse perspectives to the framework development, we acknowledge that we have not developed a framework that considers all cultural needs and sensitivities, and that specific co‐design practices and frameworks may be needed when working with particular groups, for example Aboriginal and Torres Strait Islander Peoples and communities. We also acknowledge that our co‐design team predominantly included only two individuals and, whilst supported by a diverse steering committee, the development process may have benefited from additional expertise within the core co‐design team. Importantly, as data were collected to inform framework development rather than for comprehensive qualitative inquiry, it is not possible to present quotations or undertake a more extensive qualitative analysis; consequently, the findings are presented as features and design decisions incorporated into the framework rather than as independently derived themes.

There are also limitations to the framework itself. As a practical framework designed for health service settings, there is a risk that users may interpret the co‐design process as more linear and predictable than it is in practice. While the framework attempts to acknowledge the iterative and relational nature of co‐design, no framework can fully capture the complexity, flexibility and context‐specific adaptations required during real‐world co‐design projects. We therefore propose this framework as a starting point for co‐designers, with gentle reminders and reassurance about the relational and iterative aspects of the process. The framework was developed within a large Australian public health service and therefore reflects the structures, governance requirements and organisational culture of that setting. Although we anticipate broad applicability, adaptation may be required when applying the framework in other health systems, sectors or cultural contexts. Finally, the effectiveness of the framework in supporting co‐design practice and building co‐design capability has not yet been comprehensively evaluated. This would be an important focus of future research, guided by new co‐design evaluation frameworks [45], across case studies both within and beyond our health service.

## 8 Conclusions

Through a co‐design process, we have developed a framework for health services co‐design that is freely available online for use by anyone who is new to co‐design. It is a framework developed by and for consumers, as well as health services staff and researchers, and has a deliberate focus on balancing power, inclusion and equity, trauma‐informed design, and bringing together people with diverse lived and professional experience to solve problems in healthcare.

## Author Contributions

**Adrienne M. Young:** conceptualisation, investigation, funding acquisition, writing – original draft, methodology, visualisation, validation, writing – review and editing, project administration. **Anja Christoffersen:** conceptualisation, investigation, writing – original draft, methodology, validation, visualisation, writing – review and editing, project administration. **Dale Trevor:** investigation, methodology, writing – review and editing. **Suzanne Stringer:** investigation, methodology, writing – review and editing. **Tim Cudmore:** investigation, methodology, writing – review and editing. **Leonie Sanderson:** investigation, methodology, writing – review and editing. **Gillian Nasato:** investigation, methodology, writing – review and editing. **Lisa Anemaat:** investigation, methodology, validation, writing – review and editing. **Katrina L. Campbell:** investigation, methodology, writing – review and editing.

## Conflicts of Interest

The authors declare no conflicts of interest.

## Supporting information


Supporting File

## Data Availability

The data that support the findings of this study are available on request from the corresponding author. The data are not publicly available due to privacy or ethical restrictions.

## Footnotes

^a^
Expert mindset: a mindset of believing you have the knowledge and/or experience to be the expert on the ‘way things should be done' [17].

## References

[1] C. Vargas , C. Zorbas , G. R. Longworth , et al., “Exploring Co‐Design: A Systematic Review of Concepts, Processes, Models, and Frameworks Used in Public Health Research,” Journal of Public Health 47, no. 4 (2025): e616–e639. 40796276 10.1093/pubmed/fdaf084 PMC12669994

[2] Australian Commission on Safety and Quality in Health Care . Partnering with Consumers Standard. 2024, accessed 29th October 2024.

[3] V. J. Palmer , W. Weavell , R. Callander , et al., “The Participatory Zeitgeist: An Explanatory Theoretical Model of Change in An Era of Coproduction and Codesign in Healthcare Improvement,” Medical Humanities 45, no. 3 (2019): 247–257. 29954854 10.1136/medhum-2017-011398 PMC6818522

[4] D. M. Berwick , “Era 3 for Medicine and Health Care,” Journal of the American Medical Association 315, no. 13 (2016): 1329–1330. 26940610 10.1001/jama.2016.1509

[5] World Health Organization . Human rights. 2023, accessed 29th October 2024.

[6] L. K. Wiles , D. Kay , J. A. Luker , et al., “Consumer Engagement in Health Care Policy, Research and Services: A Systematic Review and Meta‐Analysis of Methods and Effects,” PLoS One 17, no. 1 (2022): e0261808. 35085276 10.1371/journal.pone.0261808 PMC8794088

[7] L. A. Baumann , A. K. Reinhold , and A. L. Brütt , “Public and Patient Involvement in Health Policy Decision‐Making on the Health System Level – A Scoping Review,” Health Policy 126, no. 10 (2022): 1023–1038. 35918211 10.1016/j.healthpol.2022.07.007

[8] P. Sullivan‐Taylor , E. Suter , S. Laxton , N. D. Oelke , and E. Park , “Integrated People‐Centred Care in Canada ‐ Policies, Standards, and Implementation Tools to Improve Outcomes,” International Journal of Integrated Care 22, no. 1 (2022): 8. 10.5334/ijic.5943 PMC8815438 35136389

[9] Health and Social Care Act . 2001, accessed 29th October 2024.

[10] É. Ní Shé and R. Harrison , “Mitigating Unintended Consequences of Co‐Design in Health Care,” Health Expectations 24, no. 5 (2021): 1551–1556. 34339528 10.1111/hex.13308 PMC8483209

[11] Auckland Co‐design Lab . Co‐design Capability and Conditions Framework. 2018, accessed 29th October 2024.

[12] The Point of Care Foundation . Experience‐based co‐design toolkit. 2013, accessed 29th October 2024.

[13] Picker Institute Europe . 4. Getting started and building the project team. 2026.

[14] Picker Institute Europe . 9. Editing the film. 2026.

[15] S. Bowen , K. McSeveny , E. Lockley , D. Wolstenholme , M. Cobb , and A. Dearden , “How Was It for You? Experiences of Participatory Design in the Uk Health Service,” CoDesign 9, no. 4 (2013): 230–246.

[16] D. Masterson , K. Areskoug Josefsson , G. Robert , E. Nylander , and S. Kjellström , “Mapping Definitions of Co‐Production and Co‐Design in Health and Social Care: A Systematic Scoping Review Providing Lessons for the Future,” Health Expectations 25, no. 3 (2022): 902–913. 35322510 10.1111/hex.13470 PMC9122425

[17] K. A. McKercher , Beyond Sticky Notes. Doing co‐design for Real: Mindsets, Methods, and Movements (Beyond Sticky Notes, 2020). 1st ed..

[18] Design Justice Network . Design Justice Network Principles. 2018, accessed 29th October 2024.

[19] S. Staniszewska , J. Brett , I. Simera , et al., “GRIPP2 Reporting Checklists: Tools to Improve Reporting of Patient and Public Involvement in Research,” BMJ 358 (2017): j3453. 28768629 10.1136/bmj.j3453 PMC5539518

[20] International Association for Public Participation . IAP2 Public Participation Spectrum. 2018, accessed 29th October 2024.

[21] R. Dietkus , “The Call for Trauma‐Informed Design Research and Practice,” Design Management Review 33, no. 2 (2022): 26–31.

[22] S. Elo and H. Kyngäs , “The Qualitative Content Analysis Process,” Journal of Advanced Nursing 62, no. 1 (2008): 107–115. 18352969 10.1111/j.1365-2648.2007.04569.x

[23] G. Harvey and A. Kitson , “Parihs Revisited: From Heuristic to Integrated Framework for the Successful Implementation of Knowledge into Practice,” Implementation Science: IS 11, no. 1 (2016): 33. 27013464 10.1186/s13012-016-0398-2 PMC4807546

[24] K. Anderson , A. Gall , T. Butler , et al., “Development of Key Principles and Best Practices for Co‐Design in Health With First Nations Australians,” International Journal of Environmental Research and Public Health 20, no. 1 (2022): 147. 36612467 10.3390/ijerph20010147 PMC9819583

[25] Metro North Health . Co‐designed patient videos provide accessible nutrition information for patients. 2024, accessed 19th December 2024.

[26] C. E. Queensland Metro North Health Telehealth Co‐design Project. 2024; 29th August 2025.

[27] N. Meloncelli , S. A. Wilkinson , A. Rushton , et al., “Co‐Designing The Healthy Gut Diet for Preventing Gestational Diabetes: Co‐Design Methods and Process Outcomes,” Journal of Human Nutrition and Dietetics 37, no. 5 (2024): 1231–1244. 38894634 10.1111/jhn.13341

[28] Consumer Health Partnerships . Episode 2: Partnering for Better Health: Co‐Design Strategies. 2024, accessed 19th December 2024.

[29] Patient Experience Agency . The Fundamentals of Co‐design in Healthcare. 2024, accessed 19th December 2024.

[30] Beacon Strategies . Co‐design in Healthcare: a comprehensive guide. 2024, accessed 19th December 2024.

[31] Queensland Health . *Consumer and Carer Engagement Framework*: Office of the Chief Psychiatrist. 2024.

[32] NSW Government . Library of co‐design resources. 2025, accessed 2nd October 2025.

[33] T. Greenhalgh , L. Hinton , T. Finlay , et al., “Frameworks for Supporting Patient and Public Involvement in Research: Systematic Review and Co‐Design Pilot,” Health Expectations 22, no. 4 (2019): 785–801. 31012259 10.1111/hex.12888 PMC6737756

[34] C. Vargas , C. Zorbas , G. R. Longworth , et al., “Exploring Co‐Design: A Systematic Review of Concepts, Processes, Models, and Frameworks Used in Public Health Research,” Journal of Public Health (Oxford, England) 47, no. 4 (2025): e616–e639. 40796276 10.1093/pubmed/fdaf084 PMC12669994

[35] R. Essex , J. Kennedy , D. Miller , and J. Jameson , “A Scoping Review Exploring the Impact and Negotiation of Hierarchy in Healthcare Organisations,” Nursing Inquiry 30, no. 4 (2023): e12571. 37338510 10.1111/nin.12571

[36] B. Scholz , J. Bocking , C. Platania‐Phung , M. Banfield , and B. Happell , “Not An Afterthought”: Power Imbalances in Systemic Partnerships Between Health Service Providers and Consumers in a Hospital Setting,” Health Policy 122, no. 8 (2018): 922–928. 30017107 10.1016/j.healthpol.2018.06.007

[37] Y. Huo , L. Couzner , T. Windsor , K. Laver , N. N. Dissanayaka , and M. Cations , “Barriers and Enablers for the Implementation of Trauma‐Informed Care in Healthcare Settings: A Systematic Review,” Implementation Science Communications 4, no. 1 (2023): 49. 37147695 10.1186/s43058-023-00428-0 PMC10161455

[38] A. R. Gagliardi , W. Berta , A. Kothari , J. Boyko , and R. Urquhart , “Integrated Knowledge Translation (Ikt) in Health Care: A Scoping Review,” Implementation Science: IS 11 (2016): 38. 26988000 10.1186/s13012-016-0399-1 PMC4797171

[39] D. Banner , M. Bains , S. Carroll , et al., “Patient and Public Engagement in Integrated Knowledge Translation Research: Are We There Yet?,” Research Involvement and Engagement 5, no. 1 (2019): 8. 30805202 10.1186/s40900-019-0139-1 PMC6373045

[40] T. L. Dimopoulos‐Bick , C. O'Connor , J. Montgomery , et al., “Anyone Can Co‐Design?”: A Case Study Synthesis of Six Experience‐Based Co‐Design (Ebcd) Projects for Healthcare Systems Improvement in New South Wales, Australia,” Patient Experience Journal 6, no. 2 (2019): 93–104.

[41] F. O'Donoghue , M. T. O'Donnell , T. P. Griffin , E. Fahy , E. Newell , and A.‐M. Creaven , “Enablers and Barriers to An Experience‐Based Co‐Design Process to Develop Service Improvements in Enhanced Community Care in Ireland: A Qualitative Study,” Health Expectations 28, no. 2 (2025): e70206. 40072255 10.1111/hex.70206 PMC11898217

[42] M. Ramos , S. Bowen , P. C. Wright , M. G. G. Ferreira , and F. A. Forcellini , “Experience Based Co‐Design in Healthcare Services: An Analysis of Projects Barriers and Enablers,” Design for Health 4, no. 3 (2020): 276–295.

[43] R. Cox , M. Molineux , M. Kendall , B. Tanner , and E. Miller , “Co‐Produced Capability Framework for Successful Patient and Staff Partnerships in Healthcare Quality Improvement: Results of a Scoping Review,” BMJ Quality & Safety 31, no. 2 (2022): 134–146. 10.1136/bmjqs-2020-012729 PMC8784995 34253682

[44] L. Anemaat , D. A. Copland , V. J. Palmer , and S. J. Wallace , “Co‐Designing Aphasia Services: Evaluation of Involvement and Processes to Support Inclusion of People With Post Stroke Aphasia,” Health Expectations 28, no. 4 (2025): e70372. 40783948 10.1111/hex.70372 PMC12335853

[45] S. Peters , L. Guccione , J. Francis , et al., “Evaluation of Research Co‐Design in Health: A Systematic Overview of Reviews and Development of a Framework,” Implementation Science 19, no. 1 (2024): 63. 39261956 10.1186/s13012-024-01394-4 PMC11391618

