# Supplementary material for: Better Healthcare Together: Co‐Designing a Framework for Health Service Co‐Design
Source: Health Expect. 2026 Jul 28;29(4):e70784. doi: 10.1111/hex.70784 (PMC13411800; doi:10.1111/hex.70784)
Supplement: Supplementary file 1 — Supporting File [file HEX-29-e70784-s001.docx]

**Supplementary Materials 1 – GRIPP2 Short form^1^**

| **Section and topic** | **Item** | **Reported on Page no.** |
| --- | --- | --- |
| 1: Aim | Report the aim of PPI in the study | P. 4 |
| 2: Methods | Provide a clear description of the methods used for PPI in the study | P. 7-9 |
| 3: Study results | Outcomes—Report the results of PPI in the study, including both positive and negative outcomes | P. 12-19 |
| 4: Discussion and conclusions | Outcomes—Comment on the extent to which PPI influenced the study overall. Describe positive and negative effects | P. 19-21 |
| 5: Reflections/critical perspective | Comment critically on the study, reflecting on the things that went well and those that did not, so others can learn from this experience | P. 22-23 |

**References**

**1.** Staniszewska S, Brett J, Simera I, Seers K, Mockford C, Goodlad S, et al. GRIPP2 reporting checklists: tools to improve reporting of patient and public involvement in research. *BMJ.* 2017;358:j3453.

**Supplementary Materials 2 – Identified barriers and enablers to co-design, mapped to the i-PARIHS framework^1^**

***Characteristics of the intervention of co-design***

| **Factor** | **Source** | **Addressed within framework** | **Additional strategies required** |
| --- | --- | --- | --- |
| Confusion about what is co-design, intangible and complex ideas  *Clarity* | Health service staff  Consumers | Clear definition, starting point to direct to external resources  Case studies to demonstrate scope | Pictures and videos to help paint a picture of what co-design looks and feels like |
| Potential to be perceived as overly complex (or over complicated), can only be done as a big project  *Relative advantage, trialability* | Health service staff  Consumers | Layered to provide foundation and more advanced content, feedback and refinement to ensure framework simplifies rather than confuses, lay language  Case studies to demonstrate application | Mentoring via community of practice  Co-design showcase to highlight a range of projects |
| Difficult to state a clear ‘business case’ for co-design  *Relative advantage; underlying evidence* | Health service staff | “Case for co-design” section including links to strategy, standards and evidence | Modify funding criteria to promote co-design  Coordinated evaluation of co-design to demonstrate the value to a range of stakeholders |
| Discomfort with non-linear and iterative process  *Degree of novelty* | Health service staff  Consumers  Authors’ experience | Present process as non-linear journey, attributes and mindset to encompass the need for flexibility  Case studies to show flexibility and changing mindset | Mentoring via community of practice |
| Often an emphasis on the outcome, rather than the collaboration process or mindset  *Degree of novelty* | Health service staff  Consumers  Authors’ experience | Co-design mindset and conditions as key part of co-design; evaluation section includes evaluation of the co-design process, not just the outcomes. | Co-design showcase to highlight partnership outcomes |
| Opportunity to integrate into quality improvement, and research processes  *Degree of fit* | Health service staff | Not addressed | Embed co-design within quality improvement and research policies, processes, training, websites and funding schemes |

**Individuals involved in or affected by co-design**

| **Factor** | **Source** | **Addressed within framework** | **Additional potential strategies** |
| --- | --- | --- | --- |
| May not feel safe or supported to share lived experience (consumers, staff)  *Power and authority, professional boundaries* | Health service staff  Consumers | Engaging the lived experience (including from staff), power dynamics and physical space for co-design  Trauma informed approach to co-design | Mentoring via community of practice  Role-play and modelling (seeing other co-design in action)  Videos talking about what a safe space feels like from the consumer and staff perspective  Consumer-focused community of practice or peer-support initiatives  Availability of psychosocial supports for consumers (similar to employee assistance programs) |
| Power imbalance in decision making and contribution (consumers and staff, as well as within consumer groups and staff groups)  *Power and authority* | Health service staff  Consumers  Authors’ experience | Roles and accountabilities, focus on equity, recommendation to form consumer caucus or group prior to co-design, rules of engagement, inviting staff to share lived experience if safe to do so, time and funding (before co-design) to educate consumers on budgetary/project reporting parts of co-design  Co-design team including a consumer co-lead, focus on equity  Example ‘terms of reference’ that focus on contributions/ expectations rather than positions | Dismantle norms that reinforce hierarchy (e.g. stating professional/position titles in introductions) |
| Knowledge about the co-design process and methods  *Skills and knowledge* | Health service staff  Consumers | Co-design process section of framework  Case studies to demonstrate application of the knowledge | Formalised relationships with external design experts |
| Knowledge about how to engage “the right consumers”  *Skills and knowledge* | Health service staff  Consumers | Engaging the lived experience, the co-design team to provide outline of composition and attributes, peer performance review to ensure the right fit once engaged  Example ‘expression of interest’ template for co-design team members | Mentorship  Organisational processes and relationships to connect with diverse communities  Organisational processes to support place-based engagement to enable connection with ‘hard to reach’ communities |
| Lack of diversity within the team, reliance on the same consumers  *Skills and knowledge; values and beliefs* | Health service staff  Consumers  Authors’ experience | Co-design team, inclusive mindset and engaging lived experience; also about accessible spaces and flexible engagement so not to exclude people, equity in co-design | Organisational processes and relationships to connect with diverse communities |
| Requires relationships between individuals in the co-design team  *Collaboration and teamwork* | Health service staff  Consumers  Authors’ experience | Include first step about getting to know each other, inclusion of ice-breakers/methods to get to know each other | Mentoring via community of practice  Mindshift to valuing relationship building as legitimate work  Role-play and modelling (seeing co-design in action) |
| Expert mindset, ego, personal agendas  *Values and beliefs, power and authority* | Health service staff  Consumers  Authors’ experience | Fundamentals of co-design and attributes of co-design team members, peer performance review |  |
| Unrealistic expectations of the time it takes to change health systems  *Time, resources, support* | Health service staff  Consumers  Authors’ experience | Expectations for co-design | Change in funding/ project timelines to allow adequate time for co-design; Provide implementation support and funding after the co-design phase has completed; Co-design showcase to show impact and timeframes to achieve this |
| Not adequately recognised for their contribution  *Time, resources, support; collaboration and teamwork* | Consumers | Payment and recognition in other ways (presentations, awards, named on reports etc)  Example budget template to promote timely payments | Co-design showcase to give recognition to co-designers  Funding to support consumers to attend and present at conferences/ forums |
| Participants are not informed of the outcomes of co-design  *Collaboration and teamwork* | Consumers | A specific step related to “Share and Celebrate”, roles and accountabilities to include feedback loops, engaging with lived experience (follow-up and follow through) | Co-design showcase open to all people to hear outcomes of projects  Organisational funding and processes to hold community events |
| No mechanism to manage the performance of the co-design team and leads  *Collaboration and teamwork* | Consumers | Clear roles and accountabilities  Peer performance review concept and tool |  |
| Low health literacy of consumers  *Knowledge and skills* | Health service staff  Consumers | Focus on skill building and learning | Consumers supported to access a range of learning opportunities |
| Genuine and increasing interest from staff and consumers  *Motivation* | Authors’ experience | Framework available for all to use | Mentoring via community of practice  Establish a champion network, mailing list  Create opportunities to involve staff in co-design as participants  Co-design showcase |
| Requires significant project management (time, templates)  *Time, resources, support* | Authors’ experience | Before you start co-design (funding and time), roles and accountabilities, tools to assist with managing budgets, co-design project plan template and checklists? |  |

**Inner context of the health service**

| **Factor** | **Source** | **Addressed within framework** | **Additional potential strategies** |
| --- | --- | --- | --- |
| Complexity and visibility of the payment process for engagement for staff and consumers  *Structure and systems* | Consumers  Authors’ experience | Not addressed | Simplify payment process, develop resources to communicate process to consumers and business managers |
| Payment process not fit for purpose for consumer co-lead roles  *Structure and systems* | Authors’ experience | Not addressed | Redesign payment system  Develop policies to pay consumers as consultant and/or casual staff member, as well as via honorarium/ gift cards |
| Recruitment process not fit for purpose for consumer co-lead roles  *Structure and systems* | Authors’ experience | Example ‘expression of interest’ template for co-design team members | Expand the scope of consumer roles and processes within the health service |
| Power dynamic of staff ‘choosing’ consumers – concern about choosing those that are more aligned with the health service, rather than those who will challenge  *Structure and systems* | Consumers | Not addressed | Transparent and inclusive processes and guidelines to support consumer recruitment  Organisational processes and relationships to connect with diverse communities |
| Inadequate/ lack of funding, no mechanism for funding co-design itself  *History of innovation and change Leadership support* | Health service staff | Providing advice on what the co-design budget should include, realistic recommendations around consumer time commitment | Dedicated funding rounds to support early consumer and community engagement in project ideation phase  Dedicated funding within organisational units/ teams to support consumer engagement |
| Lack of understanding/ support from “middle managers” but support at highest level in MN  *Leadership support* | Health service staff  Consumers | Include “Case for co-design”, with generic points about the benefits that could be used in business cases; recommend inclusion of manager in the co-design team | Co-design showcase with specific invitation/ incentive for “middle managers” to attend  Integrate in co-design principles into leadership/ development courses |
| Tendency to emphasise tangible outcomes, rather than skill building and mindset/culture  *Organisational priorities* | Health service staff  Consumers | Evaluation to include focus on the process, and encourage collaborative evaluation  Case for co-design to include the outcome of trust and skill building of consumers and staff | Co-design showcase celebrating the process, skill building and mindset, as well as outcomes. |
| No way for consumers or front-line staff to put forward their ideas for co-design or contribute to prioritisation  *Culture* | Consumers | Not addressed | Mechanism for service priorities and project ideas to be submitted by consumers/ public  Public voting on short-listed projects prior to allocating funding |
| Lack of creativity and inability to see new ways of doing, strong status quo  *Culture* | Health service staff  Consumers | Recommends “External perspective” as key part of co-design team, links to creative methods | Mentoring in creative methods via community of practice  Formalised relationships with design experts |
| No clear “home” for co-design in the organisational structure  *Mechanism for embedding change* | Authors’ lived experience | Not addressed | Establish cross-departmental governance and working groups |
| Regular reporting of co-design projects and outcomes at the executive level  *Evaluation and feedback processes* | Authors’ lived experience | Not addressed | Co-design showcase  Dedicated consumer engagement awards within staff excellence, research, quality improvement awards  Place co-design on the executive board meeting agenda |

**Outer setting of Queensland and Australian health, social and political system**

| **Factor** | **Source** | **Addressed within framework** | **Additional potential strategies** |
| --- | --- | --- | --- |
| Support for co-design at state level, broader community  *Incentives and mandates*  *Inter-organisational networks and relationships* | Consumers | Not addressed | Public campaign on consumer engagement and co-design  Integrate framework into statewide training and resources |
| Required by standards  *Regulatory frameworks* | Health service staff  Consumers | Highlighted in ‘case for co-design’ as an incentive | Organisational policies and support for consumer leadership and priority setting to avoid tokenism/ tickbox |

**Facilitation**

| **Factor** | **Source** | **Addressed within framework** | **Additional potential strategies** |
| --- | --- | --- | --- |
| Skills that are developed through mentoring, reflection and lived experience of co-design  *Facilitator journey* | Health service staff  Consumers  Authors’ lived experience | Not addressed | Mentoring via community of practice  Role-play and modelling (opportunities to practise and seeing other co-design in action)  Offer training in facilitation skills |
| Need for impartial (and sometimes external) facilitator  *Conflict management and resolution* | Health service staff  Consumers  Authors’ lived experience | Clear roles and expectations of facilitator  Inclusion of an external facilitator prompt in the ‘Fundamentals of Co-design’ questionnaire | Create a peer network of co-designer that could facilitate for each other  Access to and funding for external facilitators |

**References**

**1.** Harvey G, Kitson A. PARIHS revisited: from heuristic to integrated framework for the successful implementation of knowledge into practice. *Implement Sci.* 2016;11(1):33.
